# Altered oral microbiome, but normal human papilloma virus prevalence in cartilage-hair hypoplasia patients

**DOI:** 10.1186/s13023-024-03164-3

**Published:** 2024-04-18

**Authors:** Heidi Arponen, Svetlana Vakkilainen, Natalie Tomnikov, Teemu Kallonen, Steffi Silling, Outi Mäkitie, Jaana Rautava

**Affiliations:** 1 Department of Oral and Maxillofacial Diseases, Helsinki University Hospital Head and Neck Center, University of Helsinki, Haartmaninkatu 1, Helsinki, Finland; 2 Pediatric Research Center, Children’s Hospital, University of Helsinki, Helsinki University Hospital, Helsinki, Finland; 3 Western Uusimaa Wellbeing Services County, Espoo, Finland; 4 Institute of Biomedicine, University of Turku, Turku, Finland; 5 Clinical Microbiology, Turku University Hospital, Turku, Finland; 6 National Reference Centre for Papilloma- and Polyomaviruses, Institute of Virology, Faculty of Medicine, University Hospital Cologne, Cologne, Germany; 7 Research Program for Clinical and Molecular Metabolism, Faculty of Medicine, University of Helsinki, Helsinki, Finland; 8 Folkhälsan Research Center, Helsinki, Finland; 9 Department of Molecular Medicine and Surgery, Karolinska Institutet and Clinical Genetics, Karolinska University Hospital, Stockholm, Sweden; 10 Department of Pathology, HUSLAB Diagnostics, Helsinki, Finland

**Keywords:** Cartilage-hair hypoplasia, Inborn errors of immunity, Human papilloma virus, Microbiome

## Abstract

**Background:**

Cartilage-hair hypoplasia (CHH) is a rare syndromic immunodeficiency with metaphyseal chondrodysplasia and increased risk of malignancy. In this cross-sectional observational study, we examined HPV status and oral microbiome in individuals with CHH. Oral brush samples were collected from 20 individuals with CHH (aged 5–59 years) and 41 controls (1–69 years). Alpha HPVs (43 types) were tested by nested PCR followed by bead-based probe hybridization. Separately, beta-, gamma-, mu- and nu- HPV types were investigated, and a genome-based bacterial microbiome sequencing was performed.

**Results:**

We found a similar alpha HPV prevalence in individuals with CHH (45%) and controls (36%). The HPV types of individuals with CHH were HPV-16 (25%), 27, 28, and 78, and of controls HPV-3, 16 (21%), 27, and 61. Beta HPV positivity and combined beta/gamma/mu/nu prevalence was detected in 11% and 11% of individuals with CHH and in 5% and 3% of the controls, respectively. Individuals with CHH differed from the controls in bacterial microbiota diversity, richness, and in microbial composition. Individuals with CHH had lower abundance of species *Mitsuokella sp000469545, Parascardovia denticolens, Propionibacterium acidifaciens, UMGS1907 sp004151455, Salinicola halophilus, Haemophilus_A paraphrohaemolyticus, Fusobacterium massiliense*, and *Veillonella parvula*, and higher abundance of *Slackia exigua*.

**Conclusions:**

Individuals with CHH exhibit similar prevalence of HPV DNA but different bacterial microbiota on their oral mucosa compared to healthy controls. This may partly explain the previously observed high prevalence of oral diseases in CHH, and regular oral examination is warranted.

**Supplementary Information:**

The online version contains supplementary material available at 10.1186/s13023-024-03164-3.

## Background

Cartilage-hair hypoplasia (CHH) is a rare autosomal recessive syndromic immunodeficiency with skeletal dysplasia [1]. CHH is caused by variants in the *RMRP* gene, encoding the untranslated RNA molecule of the mitochondrial RNA-processing endoribonuclease [2], and clinically characterized by short stature, sparse hair, and variable degree of immune dysfunction [1, 3]. CHH is exceptionally prevalent in the Amish and Finnish populations with respective incidence of 1 in 1340 and 1 in 23 000 births [3, 4]. Owing to the immunodeficiency, individuals with CHH are at increased risk of autoimmune diseases, malignancies and lung disease [5, 6]. The risk of cancer is sevenfold, predominantly expressed as non-Hodgkin’s lymphoma and basal cell carcinoma [7]. Also, single cases of fatal lip squamous cell carcinoma and vocal cord carcinoma have been described [8].

Oral cavity is part of the mucosal immune system, and as such incorporates both local oral fluid-mediated secretory and systemic immunity [9]. The etiopathogenesis of oral diseases may be linked to local, systemic, innate or adaptive immunity, to local or systemic diseases, as well as to cellular or secretory factors [10]. Oral microbiome is defined as the collective genome of microorganisms that reside in oral cavity [11]. Oral microbiome consists of a common core microbiome and a variable unique microbiome, that reflects individual’s lifestyle and distinctive physiology [11]. The risk for pathogenic bacterial infection, such as dental decay and periodontal disease, is increased in immunocompromised individuals [12, 13], and the association between oral diseases and systemic health is bidirectional [14].

Human papilloma virus (HPV) infection is a risk factor for carcinoma in the oropharyngeal, genital, and anal regions [15, 16]. Especially the high-risk (hr) HPV alpha types HPV-16 and − 18 are commonly encountered in mucosal carcinomas [17]. High prevalence of gynecologic HPV infections has been detected in females with CHH [18]. The immunological dysregulation and vulnerability to infections may predispose individuals with CHH to prolonged HPV infections [18]. Such persistence may increase their risk for carcinoma development also in the head and neck region [19]. Epidemiological studies have also revealed a close relationship between oral microbiome and tumor occurrence [20]. Oral microbial imbalance, caused by either external alterations or damaged immune function, is a potential underlying mechanism for tumorigenesis [20]. No previous study has investigated oral HPV status and bacterial microbiome in individuals with CHH. This cross-sectional observational study tested the hypothesis that individuals with CHH have higher prevalence of oral HPV and altered oral microbiota compared with the general population.

## Material and methods

The study protocol was approved by the Research Ethics Committee of the Hospital District of Helsinki and Uusimaa (HUS836/2018). Informed consent was obtained from all the participants and/or their legal guardians prior to study onset. The study conformed to STROBE Guidelines [21].

### Study participants

**Fig. 1:**
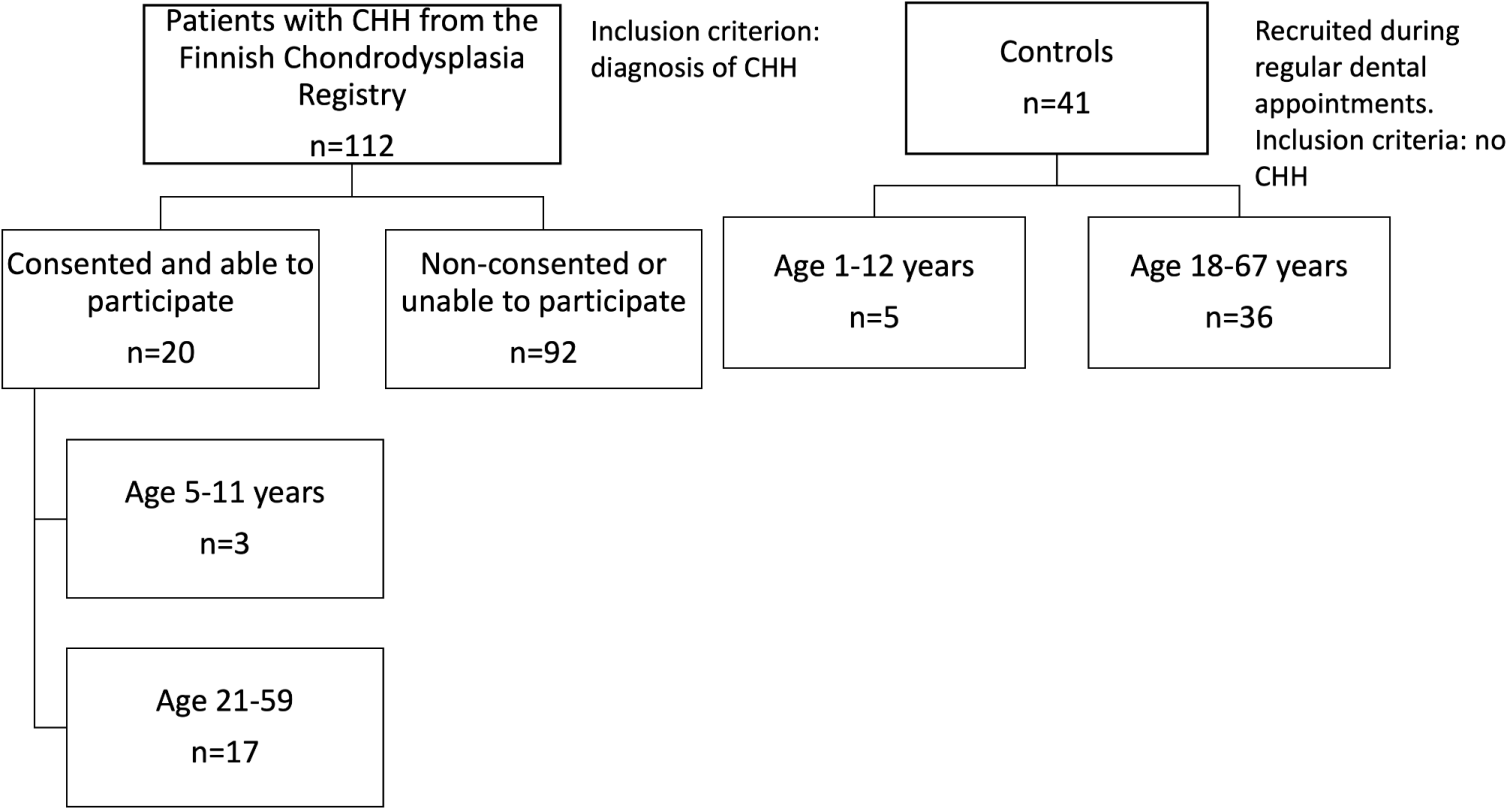
Flow chart of study subjects

All the 112 members of the Finnish Chondrodysplasia Registry were invited to participate in this study. The study coincided with the COVID-pandemic, which prevented participation of all the willing candidates due to hospital research visit policies. Eventually, 20 individuals with CHH were willing and able to attend an oral examination and brush sampling between March 2020 and May 2021. Figure 1 outlines the participant selection. None of the study participants presented with COVID-19 symptoms.

The control group consisted of a random sample of 41 volunteer patients and staff members of Espoo Municipality Dental Clinic undergoing an identical examination. A targeted ratio was two controls to one case. The HPV samples of two control individuals were lost during sample transportation and the eventual control group consisted of HPV samples of 39 individuals and oral microbiota samples of 41 individuals (Table 1).

**Table 1:** Human papilloma virus (HPV) distribution in 20 individuals with Cartilage-hair hypoplasia (CHH) and 39 controls detected with Luminex-hybridization. Positive findings are shaded

CHH	Alpha HPV	Beta HPV	Gamma, nu, my HPV
1	HPV28	Neg	Pos
2	Neg	Neg	Pos
3	Neg	Neg	Neg
4	Positive, not typeable	Neg	Pos
5	HPV78	Neg	Pos
6	Neg	Neg	Pos
7	Neg	Neg	Pos
8	HPV16	Neg	Pos
9	Neg	Neg	Neg
10	Neg	Neg	Neg
11	HPV16	Neg	Pos
12	Neg	Neg	Neg
13	Neg	Pos	Pos
14	Neg	Neg	Neg
15	Neg	Pos	Pos
16	HPV16	Neg	Pos
17	HPV16	Neg	Neg
18	HPV16	Neg	Pos
19	HPV27	Neg	Pos
20	Neg	Neg	Neg
**Controls**			
21	Neg	Neg	Pos
22	Neg	Neg	Neg
23	HPV16	Neg	Pos
24	HPV16	Neg	Pos
25	Neg	Neg	Pos
26	Neg	Neg	Neg
27	HPV16	Neg	Neg
28	HPV3	Neg	Pos
29	Neg	Neg	Pos
30	Pos, not typeable	Neg	Pos
31	Neg	Neg	Pos
32	Neg	Neg	Neg
33	Neg	Neg	Neg
34	Neg	Neg	Neg
35	Neg	Neg	Pos
36	Neg	Neg	Neg
37	Neg	Neg	Neg
38	HPV16	Neg	Neg
39	HPV16	Neg	Neg
40	HPV16	Neg	Neg
41	HPV16	Neg	Pos
42	Neg	Neg	Neg
43	Neg	Neg	Neg
44	HPV27	Neg	Pos
45	HPV27	Neg	Neg
46	Neg	Pos	Pos
47	Neg	Neg	Pos
48	Neg	Neg	Pos
49	Neg	Neg	Neg
50	Neg	Neg	Neg
51	HPV16	Neg	Neg
52	Neg	Neg	Pos
53	Neg	Neg	Neg
54	Neg	Neg	Pos
55	Neg	Neg	Pos
56	Neg	Neg	Neg
57	HPV61	Pos	Pos
58	Neg	Neg	Neg
59	HPV3	Neg	Neg

Published in conference poster at EUROGIN 2023

The oral examination included full-mouth registration of the number of active caries lesions and probing pocket depth of six sites per tooth. Pocket depth was used as an indicator of periodontal disease [22]. Clinical oral findings, smoking status, and the presence of lymphopenia and/or neutropenia of the individuals with CHH included in this study have been presented in our previous reports [1, 23]. The complete blood count was determined before the onset of the COVID-pandemic. Two oral brush samples were obtained from the buccal mucosa of both cheeks and the superior and inferior vestibular areas. The brush sample for microbiota analysis was taken with Puritan DNA/RNA collection tube Shield with Swab (Zymo Research, USA) according to manufacturer’s instructions. For HPV analysis, a Cytobrush (MedScand, Sweden) was taken and placed into 70% ethanol and frozen at − 70 °C until analyzed.

### HPV analysis

DNA extraction from Cytobrush samples was performed with high salt method [24]. The DNA extraction method has been previously used in Finnish HPV family studies, and therefore makes our findings comparable to the previous ones [25]. HPV genotypes of genus alpha were analyzed by nested PCR. The samples with positive band in gel electrophoresis were further analyzed using a bead-based Luminex system detecting the following 43 HPV types as described before [26]; hrHPV types: 16, 18, 31, 33, 35, 39, 45, 51, 52, 56, 58, 59, 68, probable/possible (p) hrHPV types: 26, 30, 53, 66, 69, 70, 73, 82, 85, 97, non-classified or low risk (lr) HPV types: 6, 11, 27, 34, 40, 42, 43, 44, 54, 55, 57, 61, 67, 71, 72, 81, 83, 84, 89, 177. HPV DNA amplification by a general primer PCR (GP5+/6+) and the subsequent detection of the products with type-specific oligonucleotide probes couples to fluorescence-labeled polystyrene bead (Luminex suspenstion array technology) (GP5+ (5’TTT GTT ACT GTG GTA GAT ACT AC-3’) (5’-biotinylated GP6+: 5’-GAA AAA TAA ACT GTA AAT CAT ATT C-3’). Any A6/A8 PCR sample that tested positive in agarose gel electrophoresis, but did not hybridize in the Luminex assay, was Sanger-sequenced. Six samples did not hybridize to one of the given probes and therefore, were sequenced using a Sanger-based technology and typed by using the NCBI Blast database (https://blast.ncbi.nlm.nih.gov/Blast.cgi). If the sequencing data was not readable, the sample was considered HPV-positive with undetermined type. All HPV16-positive samples were quantified by quantitative real-time PCR. HPV16 viral load measurement was performed using HPV16-type-specific real-time PCR on a light-cycler 480 II (Roche) [27]. This assay detects at least 50 International Units (IU) / 5 µl of HPV16 DNA. However, the nested A6/A8 HPV genotyping assay seems to be even more sensitive, as we had an HPV16-positive result but could not quantify HPV16-DNA using real-time PCR. Nested PCR protocol for the detection of genus beta HPV (EV = Epidermodysplasia verruciformis-associated HPV) was performed, but not further analyzed, as usually more than one beta-HPV type is present and sequencing does not give a clear result [28, 29]. Finally, the „FAP“ PCR was performed, detecting HPV of the genera Beta, Gamma, Mu and Nu as previously described [30]. To account for an elevated risk of contamination using nested PCR, HPV-negative cells (processing control) as well as reagent controls were included in every run. All controls were HPV-negative in every run.

### Microbiota analysis

Samples for the microbiome analysis were divided into aliquots and frozen in − 80 °C. Bacterial DNA isolation was performed with Hain GXT NA Extraction Kit using GenoXtract (Hain Lifescience GmbH, Germany) from 500 µl of sample solution. The shotgun sequencing was performed on an Illumina Novaseq 6000 instrument using 150 bp paired-end sequencing. The sequencing libraries were prepared with Nextera XT Library preparation kit. The sequencing run included ZymoBIOMICS Microbial Community DNA Standard (Zymo Research Corporation, USA) as a positive sequencing control and a negative control from DNA isolation.

The sequence data was processed with CLC Microbial Genomics Module (CLC Genomics Workbench 22.0.2, Qiagen, Denmark). Workflows “Data QC and Taxonomic Profiling” and “Merge and Estimate Alpha and Beta Diversities” were used in bioinformatics analyses. These include quality, ambiguous, and automatic adapter read-through trimming. Subsequently, sequences were mapped using Qiagen’s curated QMI-PTDB database (version 2, 2022-01) containing 60 445 reference sequences. Host genome was filtered using Genome Reference Consortium Human Build GRCh38.p13 (updated 2022-04, assembly ID: GCA_000001405.28). Alpha diversity was calculated using “Estimate Alpha and Beta Diversities” workflow with maximum depth of 400 000 and 50 sampling points in rarefaction analysis. Rarefaction value of 24 491 was used. Shannon entropy, and Chao1 diversity indices were selected to represent alpha diversity. Beta diversity calculation was performed with Bray-Curtis dissimilarity index and visualized with Principal Coordinate Analysis (PCoA). Package “ggplot2” [31] was used in visualization of principal coordinates with default settings and 0.95 as confidence limit.

### Statistical analysis

As a reference for evaluation of the power of the study, we used the reported average oral HPV point prevalence of 19.5% in Finnish adult population [32]. With 80% power (alpha 0.05) the achieved study sample size would be able to reliably detect an HPV incidence of 62% or higher in the patient group.

All cases with available data were utilized. The missing HPV samples of two control individuals were assumed to be completely at random. Chi-Square test was applied to analyze the association between HPV status (dichotomous) and individuals age (categorical), smoking or periodontal status (dichotomous), as well as presence/absence of CHH or lymphopenia (in individuals with CHH). Nonparametric Mann-Whitney U test and PERMANOVA test were used to assess microbial structure alterations for alpha and beta diversities respectively. OTU tables were used to perform differential abundance analysis with Wald test at strain and species level. FDR- corrected p-values were used in differential abundance analysis. Significance was considered for *p* < 0.05 (2-sided).

## Results

Mean age of individuals with CHH was 36 years with a range of 5–59 years (40% males), and that of controls was 33 years, range 1–67 years (32% males). The difference in age was statistically insignificant between the groups (U = 451, *p* = 0.321). Of the participants with CHH, three were children aged between 5 and 11 years, and 17 were adults (aged ≥ 18 years) (Fig. 1). Of the controls, five were children aged between 1 and 12 years, and 36 were adults.

Study participants with CHH had asymptomatic or mild clinical immunodeficiency [6]. Five participants with CHH had recurrent respiratory infections, including otitis media, sinusitis, and pneumonia. Upper (*n* = 2) and lower (*n* = 4) gastrointestinal complaints were reported by six participants with CHH, and two of them had duodenal villous atrophy of unknown etiology. Seven participants with CHH had reported a history of skin warts, four of them required multiple treatment courses. Five out of these seven individuals were positive for oral HPV in the current analysis. Basal cell carcinoma (multiple episodes) had previously been diagnosed in a single participant with CHH, who tested positive for oral HPV. None of the controls had a known immunodeficiency disease.

### HPV status

The prevalence of HPV in patients with CHH was similar to the controls. HPV was detected in oral mucosa of 70% of individuals with CHH and in 64% of controls (Table 2) (χ^2^(1, *n* = 59) = 1.00, *p* = 0.946). Alpha HPV was positive in 9/20 (45%) of individuals with CHH and 14/39 (36%) of the controls (c^2^ (1, *n* = 59) = 0.461, *p* = 0.578). The HPV types detected in individuals with CHH were HPV-16 (*n* = 5) and 27, 28, 78 in one sample each. In controls, the HPV types detected were 16 (*n* = 8), 3 (*n* = 2), 27 (*n* = 2), and 61 (*n* = 1). The sequencing data was not readable and thus no HPV-type was determined in two samples. Three samples carried HPV-16 below the limit of detection of the assay. No infection with multiple types occurred. Beta HPVs positivity was detected in 2/20 (10%) of individuals with CHH and in 2/39 (5%) of the controls. A combined presence of beta/gamma/mu/nu HPV types was found in 13/20 (65%) of individuals with CHH and in 18/39 (46%) of the controls. In total, 15% of individuals with CHH and 32% of the controls were HPV-negative. In the CHH group, no association was found between HPV and individual’s age, smoking or periodontal status, or presence/absence of lymphopenia (χ^2^ (19,20 = 27, *p* = 0.107), (1,20 = 0.205, *p* = 0.651), (1,20 = 2.95, *p* = 0.399), (1,20 = 1.29, *p* = 0.256) respectively. None of the CHH patients or controls exhibited an oral mucosal HPV-related lesion.

**Table 2:** Five most abundant phyla, genera and species among 20 individuals with Cartilage-hair hypoplasia (CHH) and 41 healthy controls. The table shows the number of the taxa present in cases, controls and all samples, as well as their median strain counts. Median count signifies median taxon count

		Individuals with CHH			Controls			All		
	Taxon	n	n %	Median count	n	n %	Median count	n	n %	Median count
Phylum	*Firmicutes*	20	100	78,828	41	100	194,809	61	100	169,190
	*Proteobacteria*	20	100	32,777	41	100	106,890	61	100	77,414
	*Actinobacteriota*	20	100	47,677	41	100	65,260	61	100	59,906
	*Bacteroidota*	20	100	9971	41	100	17,215	61	100	16,267
	*Firmicutes_C*	20	100	7309	41	100	43,385	61	100	24,056
Genus	*Streptococcus*	20	100	65833.5	41	100	160,991	61	100	158,785
	*F0040*	20	100	634	37	90.2	2007	57	93.4	1135
	*Actinomyces*	20	100	5864	41	100	23,664	61	100	16,802
	*Haemophilus_D*	19	95	3185.5	41	100	8611	60	98.4	6366
	*Haemophilus*	19	95	2315	41	100	7559	60	98.4	4828
Species	*Streptococcus pneumoniae*	20	100	48452.5	41	100	117,580	61	100	105,648
	*Actinomyces viscosus*	20	100	5101	41	100	22,918	61	100	15,391
	*F0040 sp900095835*	15	75	77	31	75.6	1655	38	62.3	633
	*Streptococcus gwangjuense*	20	100	14774.5	41	100	40,680	61	100	32,877
	*Haemophilus_D parainfluenzae_A*	19	95	3038	41	100	8268	60	98.4	5944

### Oral microbiome

The samples of patients and controls were dominated by the phyla *Firmicutes*, *Proteobacteria*, *Actinobacteriota, Bacteroidota* and *Firmicutes_C* (Table 1). The most abundant genera were *Streptococcus, F0040, Actinomyces*, *Haemophilus_D* and *Haemophilus* whilst the most abundant species were *Streptococcus pneumonia*, *F0040 sp900095835*, *Actinomyces viscosus, Steptococcus gwangjuense* and *Haemophilus_D parainfluenzae_A.* The most abundant strains were *Actinomyces viscosus*, *Prevotellaceae bacterium Marseille-P2826*, *Streptococcus pneumoniae*, *Streptococcus gwangjuense*, *Haemophilus parainfluenzae T3T1* (Table 1). No significant difference was detected at species or strain level. Read count abundances were adjusted. Out of all the sequences, 69.7% corresponded to host reads, while 1.1% were reference database matches. The reference database detected at least 558 species and 375 genera, including representation from bacteria and archaea. Average number of reads before trimming was 91998249,11 (median 87,263,078) and after trimming 91952668,38 (median 87,236,649).

**Fig. 2:**
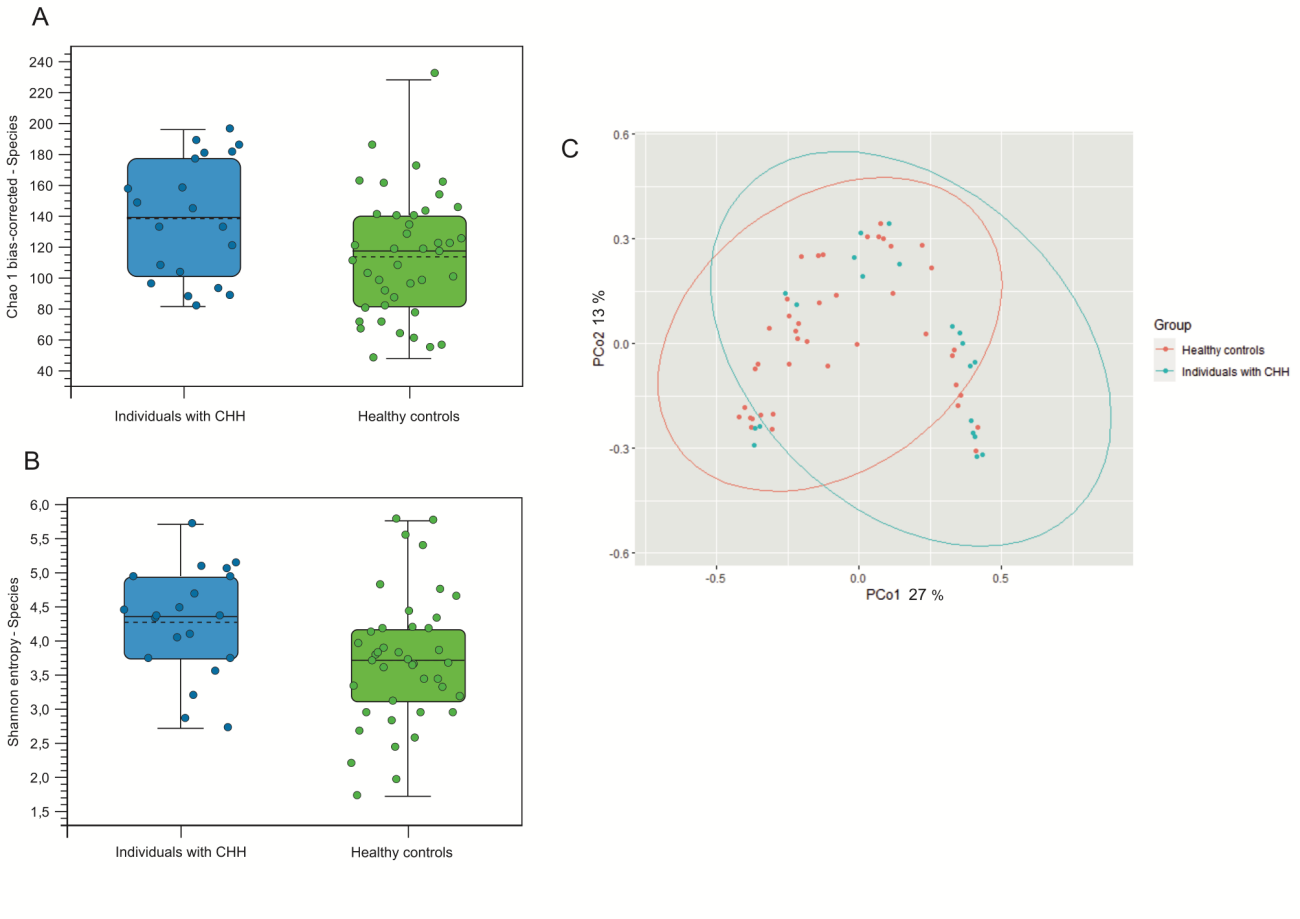
Alpha and beta diversity indices of individuals with Cartilage-hair hypoplasia and healthy controls with (A) Chao 1 bias-corrected (*p* = 0.03), (B) Shannon entropy (*p* = 0.02) metrics at species-level, and (C) Bray-Curtis (*p* = 0.01) metrics at species-level

The alpha and beta diversity metrics at species level are shown in Fig. 2. Both alpha diversity indices showed statistically significant difference between the groups, with individuals with CHH having higher alpha diversity (*p* = 0.03 with Chao 1, *p* = 0.02 with Shannon entropy). Microbial communities differed between individuals with CHH and control groups, as defined by Bray-Curtis beta diversity index (*p* = 0.01). Strain level metrics followed the same trend as species level (Additional file, Figure S1).

**Fig. 3:**
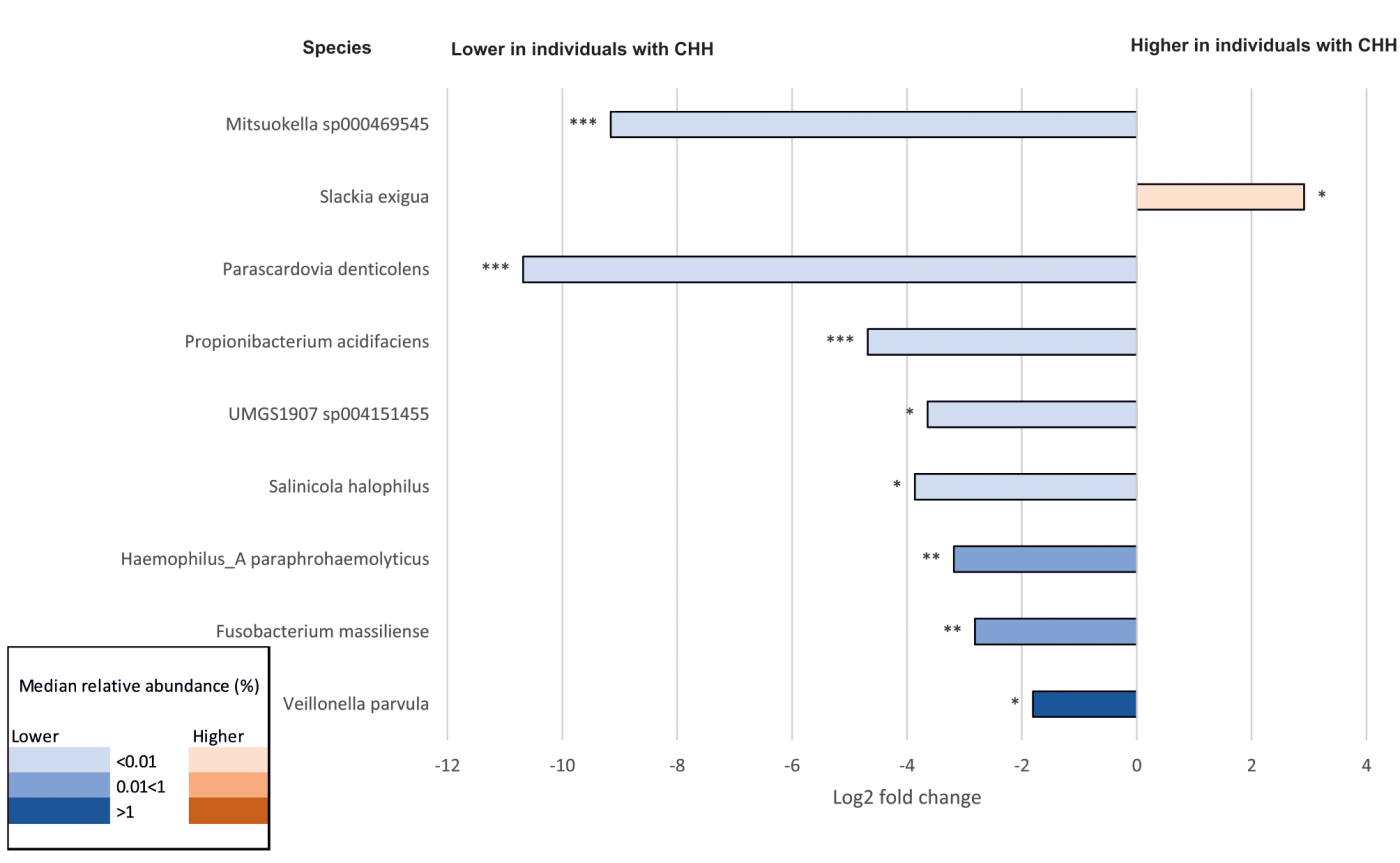
Differential abundance analysis at species level between individuals with Cartilage-hair hypoplasia and healthy controls. The data is corrected for active caries and periodontal disease. The figure contains 9 most abundant and statistically significant (FDR<0.05) species. The color of the bar indicates the median abundance of the species. Fold change indicates direction and difference of the change. *=*p*-value <0.05, **=*p*-value <0.01, ***=*p*-value <0.001

Differential abundance analysis showed significantly different bacterial species between the two groups, when corrected for active caries and periodontal disease (Fig. 3 and Additional file, Table S1). Species *Mitsuokella sp000469545, Parascardovia denticolens, Propionibacterium acidifaciens, UMGS1907 sp004151455, Salinicola halophilus, Haemophilus_A paraphrohaemolyticus, Fusobacterium massiliense*, and *Veillonella parvula* were lower in abundance among individuals with CHH, whilst *Slackia exigua* was higher (Fig. 3, Additional File, Figure S2 and Table S2).

## Discussion

To our knowledge, this is the first report on oral HPV DNA prevalence as well as oral microbiome analysis in individuals with CHH. We found that individuals with CHH exhibit similar prevalence of HPV DNA but different bacterial microbiota on their oral mucosa, compared to healthy controls.

Oral cavity hosts bacteria, viruses, and fungi that contribute to physiological and immunological functions [11]. Colonization begins at or shortly after birth, and eruption of teeth produces more surfaces for colonization [11]. Most unvaccinated individuals acquire HPV at least once in their lifetime [33]. Oral mucosa is a common site for the first exposure to HPV [34]. Despite oral HPV transmission commonly occurring through sexual contact [33], half of healthy adults demonstrate HPV-specific cell-mediated immunity, irrespective of their sexual status [35]. The infection can be acquired vertically or horizontally from the mother at an early age, resolve spontaneously, or remain latent for years [33]. In a previous Finnish HPV study on families, the parents’ incidence rate of oral mucosal HPV ranged from 8 to 34% and that of children from 9 to 23% [25, 35]. In the United States, peak prevalence of HPV infection is, in adults, at ages 30 to 34 and 60 to 64 years (7.3% and 11.4% respectively) due to incident infection, reinfection, or reactivation [36]. In our cohort, the prevalence of oral HPV was higher in the control group (64%), consisting of both children and adults, and notably higher in individuals with CHH (70%) compared with the previous Finnish HPV study.

HPV infection can be asymptomatic or display a variety of clinical manifestations [34]. The low-risk mucosal genotypes, such as HPV-6 and HPV-11, cause benign papilloma/condyloma, whereas the high-risk mucosal HPVs, such as HPV-16 and HPV-18, can cause squamous cell carcinoma in the head and neck region, especially in the oropharynx [34]. Malignant transformation requires persistent HPV infection [34]. Persistent and extensive HPV infection can result from an inadequate immune response [37]. Primary immunodeficiency, such as is associated with *EVER1, EVER2, GATA2, CXCR4*, and *DOCK8* mutations, as well as combined immunodeficiency, such as in bare lymphocyte syndrome, is associated with extensive HPV infection [38, 39]. Similarly, immunosuppression following organ transplantation or HIV infection increases the risk for HPV infection [40, 41]. In the general population, 27% of oral epithelial dysplasias harbor HPV DNA [42]. Smoking, poor oral hygiene, and old age are among factors speculated to module oral HPV persistence [33]. Previous investigations among Finnish females have shown that HPV-6 and HPV-16 are the most common genotypes in oral HPV-infections and most likely to persist [32, 35]. Our study found a higher prevalence of HPV in oral cavity of individuals with CHH than of healthy controls, although the difference did not reach statistical significance. However, as we have previously reported, there was no clinical findings suggesting HPV-related lesions on either groups [23]. From the detected HPV genotypes, high-risk HPV-16 was the most prevalent in HPV-positive individuals with CHH (56%) and controls (57%). No association between lymphopenia and oral HPV was detected in our sample possibly due to small sample size. Possible reservoirs for HPV in oral cavity include inflamed gingival pocket epithelium, ductal epithelium of the salivary glands, cryptal epithelium of the tonsils, border of oral cavity, and oropharynx [34]. A positive association between oral HPV infection and severe periodontitis has been suggested by previous studies [43], but was not detected in ours.

Previous studies have noted that the core oral microbiome of healthy individuals includes genera *Streptococcus, Actinomyces, Neisseria, Veillonella*, and *Haemophilus* [44, 45]. Our findings among the healthy controls are consistent with these previous observations. An altered microbial diversity has been found in oral fluids of individuals with common variable immunodeficiency, Wiskott-Aldrich syndrome related immunodeficiency, or immunocompromised HIV-positivity [46–48]. Periodontal manifestations are a common oral finding [49], and in individuals with Wiskott-Aldrich syndrome, species associated with periodontitis are more prevalent [48]. Similarly, we found, that oral microbial communities differ between individuals with CHH and the controls. The microbiome of individuals with CHH showed less species *Mitsuokella sp000469545*, which is identified as a periodontal pathogen, as well as *Parascardovia denticolens, Propionibacterium acidifaciens, UMGS1907 sp004151455*, and *Veillonella parvula*, that are all associated with plaque and dental decay. This may reflect the multifactorial nature of oral diseases with wide range of possible pathogenic bacteria since individuals with CHH have more frequently deep gingival pockets and decay [23]. *Slackia exigua* was highly abundant in individuals with CHH. *Slackia exigua* is an anaerobic gram-positive rod of human oral microbiota with pathogenic potential for oral and systemic infection [50–52]. Putative disease-driving pathobionts, such as *Slackia exigua*, are detected also in healthy individuals, and both genetic and environmental factors contribute to disease development [50, 53]. Thus, our finding supports the idea that immunocompromised individuals, such as CHH patients, could be at greater risk for increased pathogen colonization and translocation possibly leading to increased prevalence and severity of oral and extraoral infections. Simultaneously, it is important to note that bioinformatics analysis of microbiome data does not establish causality.

Some important limitations need to be considered when assessing our findings. The first is the possible selection bias of the patients and controls. The control group included 12 dental clinic staff members (26%) who would also be likely to maintain a good oral hygiene and mechanical plaque control thereby influencing oral microbiome, but not the HPV infection status. Due to the pandemic at the time of recruitment, CHH individuals with more severe immunodeficiency may have been more likely not to participate. Secondly, study participants could potentially have been carrying an asymptomatic subclinical COVID-infection, that might have affected oral microbiome. However, previous investigations have reported alterations in abundances of *Neisseria* as the main finding associated with COVID-19 [54]. No significant alterations in abundance of *Neisseria* were found in the present study across the groups. Thirdly, the sample size is small and thus expected to deliver results with more variance and more vulnerable to outliers. Our findings, however, provide insight for future studies, on larger cohorts, to explore the association between clinical and immunological findings.

## Conclusions

The findings of this study can be used to develop screening aimed at improving oral health of individuals with CHH. Individuals with CHH and symptomatic immunodeficiency typically suffer from recurrent respiratory tract infections [55, 56]. Lung disease and malignancies are the main cause of death among individuals with CHH [8, 57]. It has been estimated that over 12 million new cancer cases per year are caused by infectious agents, representing over 16% of all cancers [19]. Different microbiome of individuals with CHH may explain some of their risk for malignancies. Screening for potentially malignant epithelial lesions and atypical mouth ulcers is part of a regular comprehensive clinical oral examination and an aggressive diagnostic approach is recommended for individuals with CHH.

### Electronic supplementary material

Below is the link to the electronic supplementary material.

Supplementary Material 1

## Data Availability

All data supporting the findings of this study are available within the paper and its Supplementary Information. The original datasets are not publicly available due to patient privacy.

## Abbreviations

CHH: Cartilage-hair hypoplasia
hr: high-risk
HPV: Human papilloma virus
