# Supplementary material for: Altered oral microbiome, but normal human papilloma virus prevalence in cartilage-hair hypoplasia patients
Source: Orphanet J Rare Dis. 2024 Apr 18;19:169. doi: 10.1186/s13023-024-03164-3 (PMC11027548; doi:10.1186/s13023-024-03164-3)
Supplement: Supplementary file 1 — Supplementary Material 1 [file 13023_2024_3164_MOESM1_ESM.pdf]

Supplementary Figures and Tables

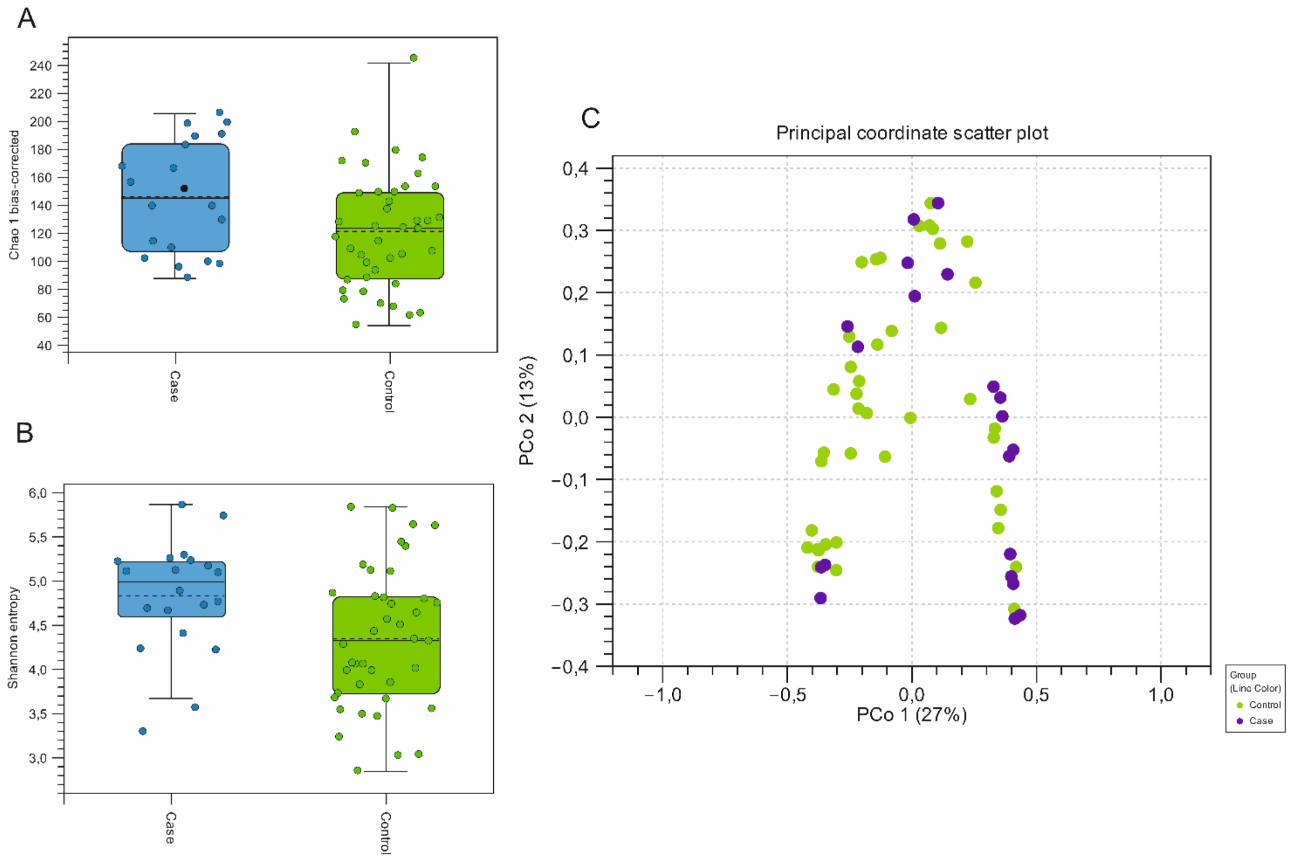

Figure S1. Alpha and beta diversity metrics of individuals with cartilage-hair hypoplasia (case) and healthy control groups at strain level. A) Chao 1 index B) Shannon entropy C) Bray-Curtis. p= 0.03 with Chao 1, p= 0.02 with Shannon entropy and p=0.01 with Bray-Curtis.

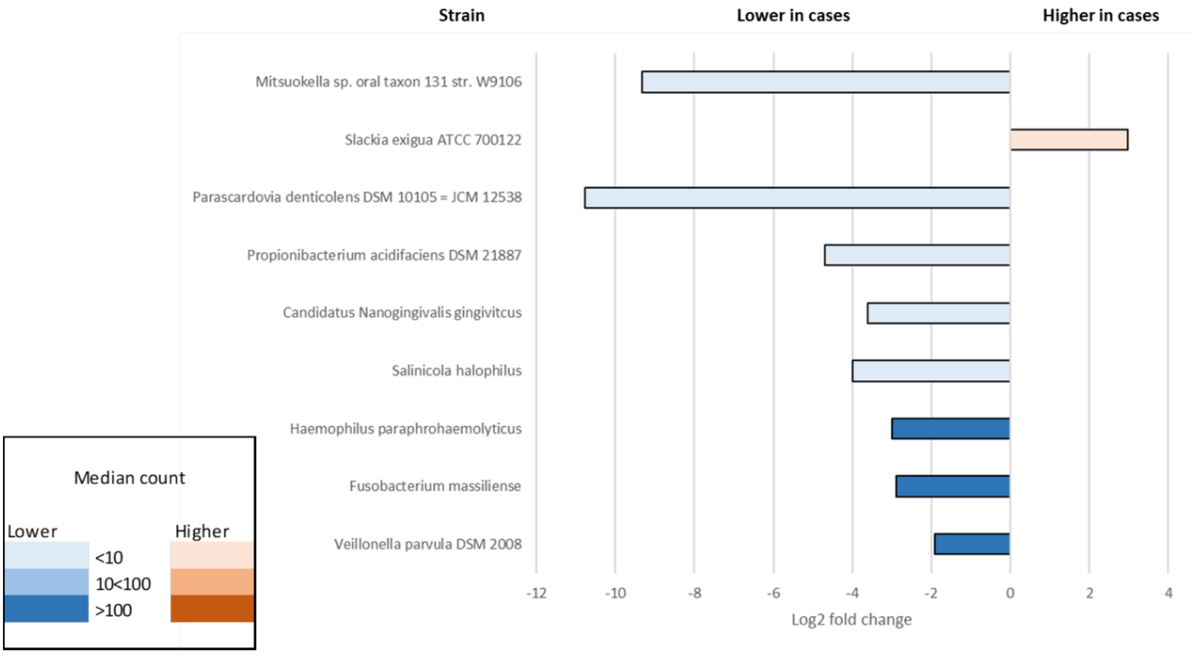

Figure S2. Differential abundance analysis at strain level between individuals with cartilage-hair hypoplasia (cases) and healthy controls. The data is corrected for active caries and periodontal disease. The figure shows 9 most abundant and statistically significant species. Median count signifies median taxon count.

Table S1. Description of the differential abundance analysis from CLC genomic workbench.

			Individuals with CHH (n=20)		Healthy controls (n=41)		All (n=61)	
Species	Log2 fold change	FDR p-value	Presence in samples (n %)	Median relative abundance (%)	Presence in samples (n %)	Median relative abundance (%)	Presence in samples (n %)	Median relative abundance (%)
Veillonella parvula	-1.808359497	0.019918712	20 (100)	0.93	40 (97.5)	1.91	60 (98,3)	1.283963647
Fusobacterium massiliense	-2.815858368	0.006040853	20 (100)	0.06	35 (85.3)	0.03	55 (90,1)	0.038114477
Haemophilus_A paraphrohaemolyticus	-3.184439745	0.004142102	17 (85)	0.05	31 (75.6)	0.04	48 (78,6)	0.044629758
Salinicola halophilus	-3.863101882	0.024422197	3 (15)	0.00	11 (26.8)	0.00	14 (22,9)	0
UMGS1907 sp004151455	-3.641708995	0.026443393	6 (30)	0.00	10 (24.3)	0.00	16 (26,2)	0
Propionibacterium acidifaciens	-4.683551871	0.000628501	7 (35)	0.00	17 (41.4)	0.00	24 (39,3)	0
Parascardovia denticolens	-10.68273845	9.30518E-09	3 (15)	0.00	6 (14.6)	0.00	9 (14,7)	0
Slackia exigua	2.912958222	0.019905129	15 (75)	0.10	15 (36.5)	0.00	30 (49,1)	0
Mitsuokella sp000469545	-9.156531814	2.28869E-07	2 (10)	0.00	5 (12.1)	0.00	7 (11,4)	0

Table S2. Detailed description of the most significant and abundant species found in this study.

Species	Log2 fold change	FDR p-value	Presence in samples (n %)	Median relative abundance (%)	Presence in samples (n %)	Median relative abundance (%)	Presence in samples (n %)	Median relative abundance (%)
Veillonella parvula	-1,808359497	0,019918712	20 (100)	0,93	40 (97.5)	1,91	60 (98,3)	1,283963647
Fusobacterium massiliense	-2,815858368	0,006040853	20 (100)	0,06	35 (85.3)	0,03	55 (90,1)	0,038114477
Haemophilus_A paraphrohaemolyticus	-3,184439745	0,004142102	17 (85)	0,05	31 (75.6)	0,04	48 (78,6)	0,044629758
Salinicola halophilus	-3,863101882	0,024422197	3 (15)	0,00	11 (26.8)	0,00	14 (22,9)	0
UMGS1907 sp004151455	-3,641708995	0,026443393	6 (30)	0,00	10 (24.3)	0,00	16 (26,2)	0
Propionibacterium acidifaciens	-4,683551871	0,000628501	7 (35)	0,00	17 (41.4)	0,00	24 (39,3)	0
Parascardovia denticolens	-10,68273845	9,30518E-09	3 (15)	0,00	6 (14.6)	0,00	9 (14,7)	0
Slackia exigua	2,912958222	0,019905129	15 (75)	0,10	15 (36.5)	0,00	30 (49,1)	0
Mitsuokella sp000469545	-9,156531814	2,28869E-07	2 (10)	0,00	5 (12.1)	0,00	7 (11,4)	0
